# Sterile water injections for analgesia in renal colic: a meta-analysis of level 1 evidence

**DOI:** 10.1007/s00345-025-05920-x

**Published:** 2025-09-16

**Authors:** Ioannis Perros, Balamrit Singh Sokhal, Christopher Swift, Mark Kitchen, Christian Mallen, Bhaskar Somani

**Affiliations:** 1 School of Medicine, Keele University, Keele, UK; 2 Royal Stoke University Hospital, University Hospital of North Midlands, Stoke-On-Trent, UK; 3 Wirral University Hospital, Liverpool, UK; 4 Department of Urology, University Hospital Southampton, Southampton, UK

**Keywords:** Meta-analysis, Renal colic, Urolithiasis, Nephrolithiasis, Analgesia

## Abstract

**Objective:**

To evaluate the effectiveness of Sterile water injections (SWIs) for acute pain relief in renal colic.

**Methods:**

This study was conducted in accordance with the Preferred Reporting items for Systematic Reviews and Meta-Analyses. MEDLINE, CINAHL, Web of Science and Cochrane were searched to identify randomised controlled trials (RCTs) comparing SWIs with placebo or other analgesics in renal colic patients. Data were pooled and analysed using random effects modelling with 95% confidence intervals (CIs).

**Results:**

Six RCTs including 1322 renal colic patients were included, with 466 (35.2%) receiving SWIs. Patients receiving SWIs had comparable demographics and presenting features to the control groups, with control medications ranging from placebos, non-steroidal anti-inflammatories, and opioids. Self-reported pain scores at 30 min following intervention were lower in SWI than placebo (MD = − 4.63, 95% CI: − 5.16, − 4.10, P < 0.001) and other analgesics (MD = − 0.36, 95% CI: − 0.52, − 0.21, P < 0.001). The use of rescue analgesia was lower in those receiving SWIs compared to placebo (OR = 0.24, 95% CI: 0.10, 0.59, P = 0.002) and other analgesics (OR = 0.46, 95% CI: 0.29, 0.74, P = 0.001). No significant side effects were attributed to SWI use.

**Conclusions:**

SWIs demonstrated superior pain relief and reduced rescue analgesia requirements, compared to placebo and standard treatment, offering a promising alternative for patients where traditional options are unsuitable.

**Supplementary Information:**

The online version contains supplementary material available at 10.1007/s00345-025-05920-x.

## Introduction

Renal colic (encompassing renal and ureteric stones) is characterised by severe pain and nausea due to kidney and ureteric stones and impacts approximately 12% of the global population at some point during their life [1–3]. The majority of kidney stones are non-infective and are linked to factors such as inadequate fluid intake, hot climates, excessive intake of protein, carbohydrates and sodium, as well as specific comorbidities, including hypertension, gout, obesity and non-alcoholic fatty liver disease [4–7]. Effective pain relief is essential for managing renal colic, with non-steroidal anti-inflammatory drugs (NSAIDs) regarded as the best analgesic options, often used in combination with opioids and paracetamol due to the typically severe pain experience [8]. Each medication class has its benefits and potential side effects.

The British Association of Urological Surgeons (BAUS) guidelines currently recommend using Ibuprofen and Diclofenac as first-line management of pain in renal colic [9]. NSAIDs are preferred due to their effectiveness in reducing pain in renal colic due to their effects on renal vasculature and urine production whilst having a lower side effect profile compared to opioids, which commonly cause nausea, vomiting, and drowsiness [2].

However, the use of NSAIDs is known to exacerbate coronary disease and increase the risk of bleeding, therefore are contra-indicated in patients with renal failure or previous upper gastrointestinal (GI) bleeds [10, 11]. Given the attributable side effects to currently used analgesics, it is prudent to explore other options. Alternative approaches such as sterile water injections (SWIs) have recently been used. These were initially explored for analgesia in labour-related back pain, which shares similar visceral and referred pain mechanisms with renal colic pain and have been referenced as far back as 1945 [12, 13]. SWIs appear to provide significant benefits in pain management and demonstrate advantages in reducing side effects compared to conventional analgesics and placebo. We note, however, that the quality of evidence varies across studies [14, 15].

There is evidence to suggest SWIs can provide significant pain relief in renal colic. However, the quality of the evidence varies, with no rigorous comparisons of SWI analgesic efficacy to NSAIDs and opioids in renal colic patients. Furthermore, there appears to be an incomplete understanding of SWI’s mechanism of action, its therapeutic efficacy, and possible adverse effects. These gaps in evidence emphasise the need for a systematic review to evaluate the efficacy of sterile water injections and compare these to conventional “standard care” analgesia for managing renal colic pain.

## Materials and methods

### Search strategy and study selection

This review protocol was registered with the International Prospective Register of Systematic Reviews (PROSPERO) with registration number CRD42023474039 and follows the Preferred Reporting Items for Systematic Review and Meta-analyses (PRISMA) guidance [16]. MEDLINE (EBSCO), Cochrane, EMBASE and Web of Science were searched from inception till June 2024. The search strategy used database subject headings, text word searches in the title and abstracts, as well as the references of included studies using terms for “Sterile water injections” and “pain relief in renal colic” (Supplementary Table S1). Ethical approval was not required, given that this was a meta-analysis of already published data.

### Inclusion and exclusion criteria

Randomised controlled trials (RCTs) that investigated the use of SWI compared to either other pain relief methods or a placebo in renal colic were considered eligible for this study. Non-randomised controlled trials, case reports, review articles, editorials, research letters, case series, case–control studies, observational studies, systematic reviews, and meta-analyses were excluded. Only articles published in English were included. Adults (> 18 years) with diagnosed urinary tract stones on imaging described the population of interest. The primary outcomes of this study included the effectiveness of pain relief, intensity of pain reduction, time to onset of pain relief, and adverse outcomes.

### Screening and data extraction

All references were imported into a reference management software, where duplicates were removed by one reviewer (BSS). Following this, two reviewers (IP and CS) independently screened the titles and abstracts of the identified studies to ensure that they met the inclusion criteria. The same two reviewers extracted data from the full-text articles into pre-formatted tables. Study characteristics such as first author, publication year, study design and journal were extracted. The results of each study were stratified into groups, correlating the use of different pain relief agents against SWIs. The results reported demographic information (number of participants, age, gender, pain intensity before intervention) as well as study outcomes (pain reduction in time intervals and use of rescue analgesia). Any disagreements throughout this process were resolved by discussion between the two reviewers, and when disagreements remained, a third reviewer provided a resolution (BSS).

### Risk of bias assessment

Each study was independently assessed, regarding its quality, by two reviewers (IP and CS) using Cochrane’s tool for RCTs (ROB2) [17]. This tool considers the risk of performance, reporting detection, attrition, and any other sources of bias in each of the included studies. Any disputes not resolved by discussion between the two reviewers were resolved by a third reviewer (BSS).

### Data analysis

Statistical analyses were performed using Review Manager 5.4 (Revman) [18]. Data were entered into Revman and confirmed accurate by an independent reviewer. Dichotomous outcomes were reported using Odds ratios (ORs), whilst for continuous outcomes, the mean difference (MD) was calculated between the groups. In instances where median and interquartile ranges were reported, these were converted into mean and standard deviation using *Hozo *et al*.’s* equation [19]. All analyses were performed with random-effects modelling. Outcomes were reported using Forest plots with 95% confidence intervals (CIs), with the unit of analysis being individual patients.

Heterogeneity between studies was investigated using the Cochran Q test, whilst I^2^ was calculated to assess the degree of heterogeneity due to the limited number of studies included. 0–50% was interpreted as insignificant heterogeneity, 50–75% was interpreted as moderate heterogeneity, and 75–100% was interpreted as large heterogeneity. A random effects model was used if there was significant heterogeneity (I^2^ > 50%), whilst a fixed-effect model was adopted if there was no significant heterogeneity.

## Results

**Fig. 1:**
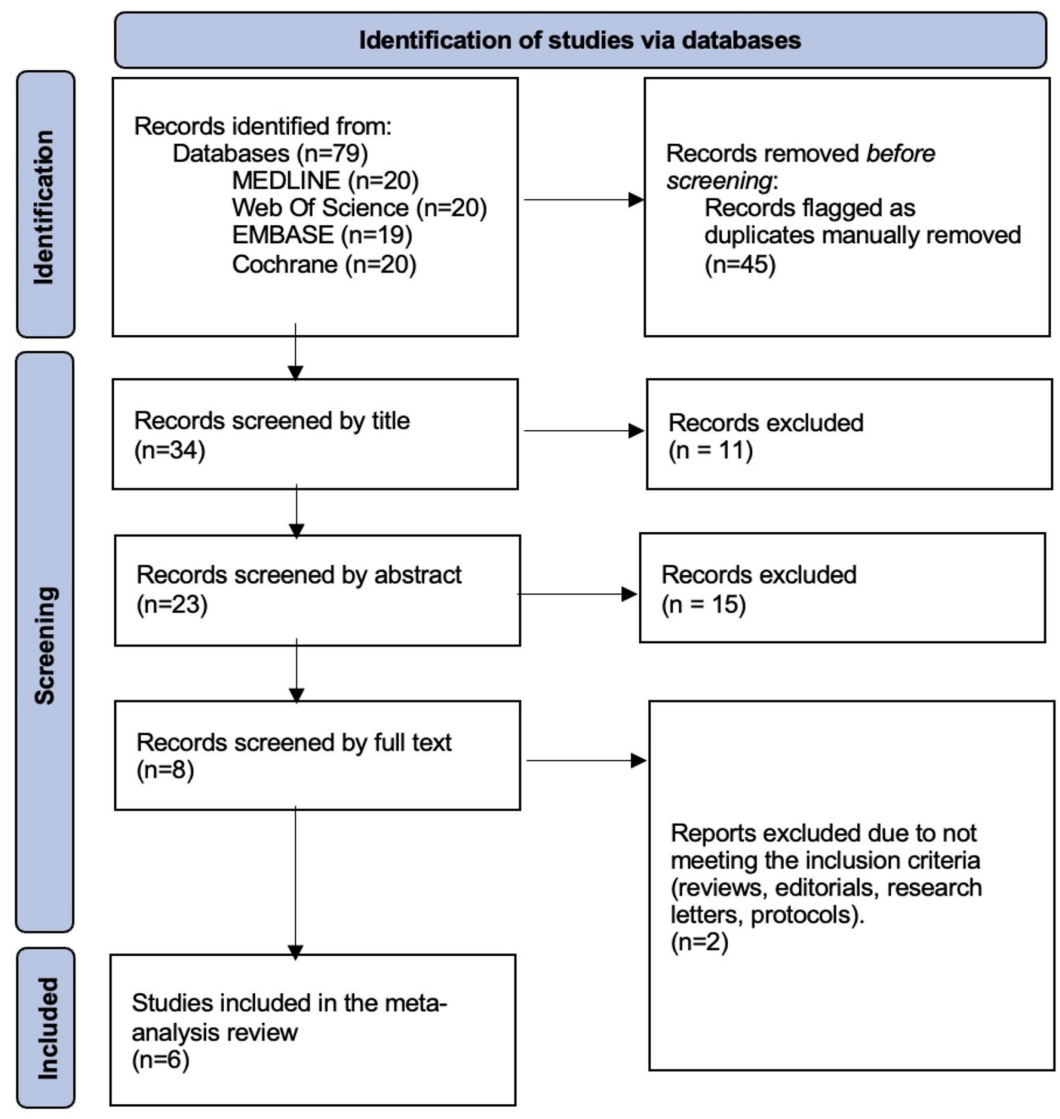
PRISMA flowchart

A total of 69 articles were retrieved, and 34 were screened against the inclusion criteria following the removal of duplicates (Fig. 1). 6 RCTs met the inclusion criteria [20–25] from 4 different countries. The trials took place between 2004 and 2023, with all studies published in English. Five trials used SWI for acute renal colic, whilst one for ongoing pain before or during extracorporeal shockwave lithotripsy (SWL). Of the above trials, three investigated SWI against Diclofenac sodium (75 mg intramuscular injection) [20–22], two against normal saline placebo [21, 23], two against paracetamol [20, 24], one against Tramadol and lastly [20], one comparing SWI plus morphine against morphine alone [25]. Details of the SWI injection protocols for each study are summarised in Supplementary Table S2. A total of 1322 patients with acute renal colic were included, with 466 of them receiving SWI for pain relief. Five studies investigated males and females, whilst one investigated renal colic in pregnant females with an unspecified gestational age. To confirm the presence of a renal calculus, all patients underwent radiological imaging with either computed tomography (CT) or ultrasound (US) scanning. Those without a stone were excluded from the original studies.

Table 1 illustrates the date of publication, country of origin, journal, study design, and sample size of the studies. Table 2 outlines the baseline characteristics of the included populations. Patients in the SWI group and the control groups were comparable in age (38.1 ± 9.8 vs 38.1 ± 10.3) and sex distribution. There was no significant difference between the pain score at presentation (7.8 ± 2.6 vs 8.1 ± 2.5), as well as stone size (13.4 ± 4.4 mm vs 13.2 ± 4.1 mm) between the SWI and control groups.

**Table 1:** Study related data

Study	Date of publication	Country	Journal	Design	Sample size (n)
Adem et al	2023	Turkey	The Journal of Emergency	RCT	320 = 80/80/80/80
Mousa et al	2020	Greece	American journal of Emergency Medicine	RCT	150 = 50/50/50
Gul et al	2020	Turkey	Urolithiasis	RCT	524 = 216/308
Ahmadnia et al	2004	Iran	Urology journal	RCT	100 = 50/50
Mozafari et al	2020	Iran	Bentham Science	RCT	98 = 49/49
Xue et al	2013	China	International urology and nephrology	RCT	45 = 21/24

*RCT* randomised controlled trial

**Table 2:** Baseline characteristics of included populations

Author	Age (years)	Female (%)	Mean stone size (mm)	VAS (/10) before treatment
Adem et al	37.6 (SD10.6) vs 38.4 (SD10.6)	66% vs 95%	3–6 mm vs 3–6 mm	8.7 (SD0.8) vs 8.6 (SD0.8)
Mousa et al	30–40 vs 30–40	Did not report	5–10 mm vs 5–10 mm	9.6(SD0.6) vs 9.7 (SD0.6)
Gul et al	41.2 (SD9.4) vs 39.9 (SD10.1)	43.5% vs 41.2%	15.6 (SD 2.3)mm vs 14.8 (SD2.8)mm	6.4 (SD2.9) vs 6.6 (SD3.2)
Ahmadnia et al	35.3 (SD9.2) vs 35.9 (SD8.9)	Did not report	7.1 (SD1.8)mm vs 7.2 (SD1.8)mm	9.9 (SD3) vs 10 (SD1.9)
Mozafari et al	33.3 (SD 7.2) vs 35.9 (SD 8.9)	Did not report	Did not report	8.1 (SD1.3) vs 9.5 (SD1)
Xue et al	27.6 (SD2.2) vs 27.2 (SD2.4)	100% vs 100%	5.7 (SD1.7)mm vs 6 (SD1.4)mm	9 (SD1) vs 8.5 (SD1)

*VAS* visual analogue scale, *SD* standard deviation

### Risk of bias assessment

The risk of bias assessment of the included studies can be found in the supplementary appendix (supplementary Figure S1). The overall quality of the studies was low. One study was rated as low risk in all seven categories [21], and one in 6 categories [20]. One exhibited unclear risk in all categories [23], whilst most did not describe their randomisation process and blinding of participants, personnel and outcome assessment, with only one study formally reporting its sample size calculation [20]. One study exhibited high risks of bias in three categories [24].

### Outcome synthesis

#### Use of rescue analgesia

**Fig. 2:**
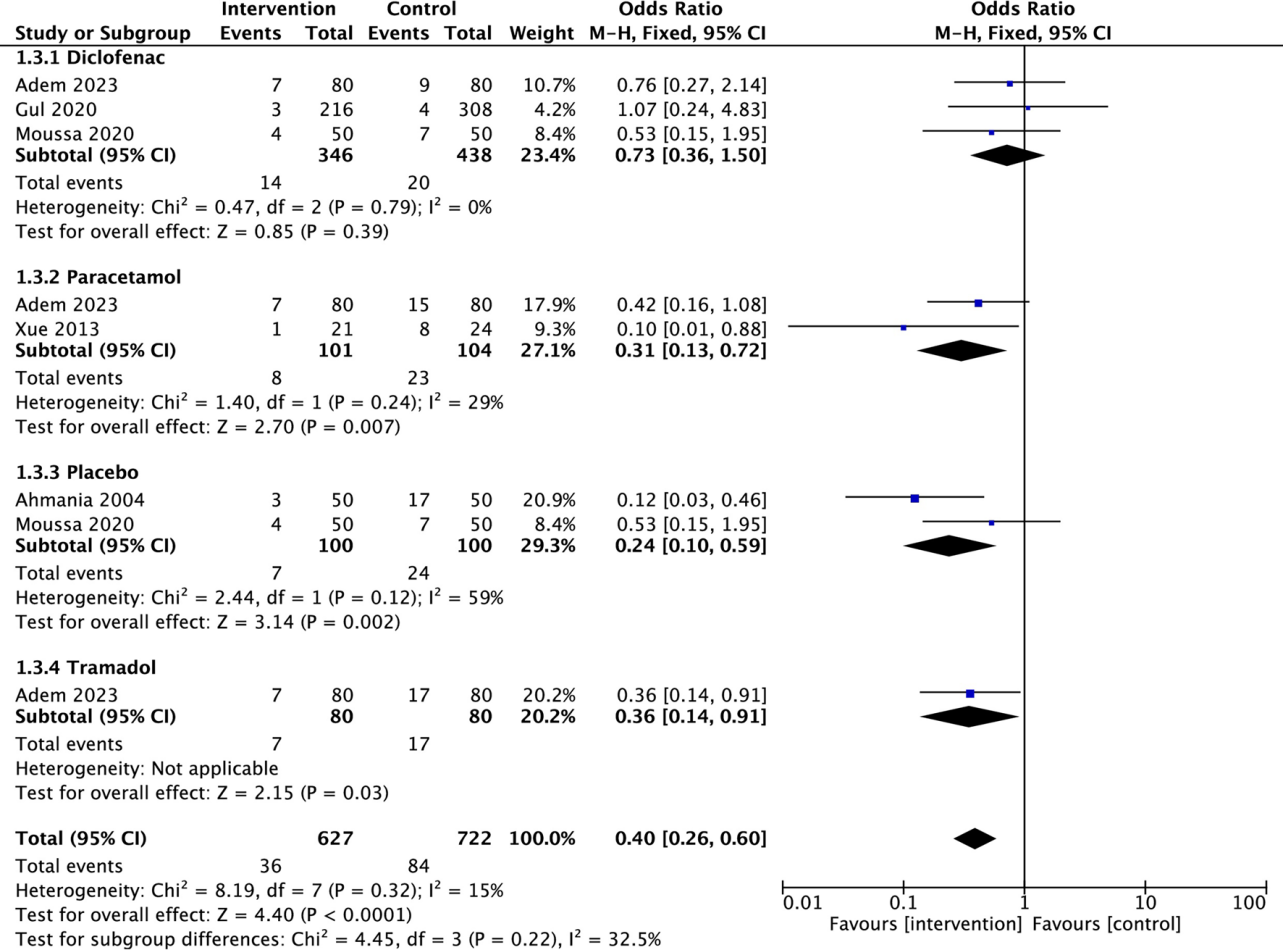
Forest plots of comparison of Sterile water injections against Diclofenac Sodium, Paracetamol, Placebo and Tramadol, rescue analgesia administered after treatment. The solid squares denote the odds ratios (ORs). The horizontal lines represent the 95% confidence intervals (CIs), and the diamond denotes the pooled effect size

Five studies investigated the use of rescue analgesia between the SWI group and the control group. The rate of rescue analgesia was notably lower in the SWI (3.9%) and Diclofenac (4.6%) groups. A significantly higher proportion of patients required rescue analgesia in the Sodium Chloride placebo group (24.0%). Similarly, the Paracetamol and Tramadol groups exhibited a higher need for additional analgesia, with 22.1% and 21.3%, respectively. Overall, SWI demonstrated a lower use of rescue analgesia than control agents (OR = 0.40, 95%CI: 0.26,0.60, P < 0.001). Between-study heterogeneity was low (I^2^ = 15%, P = 0.32). (Fig. 2).

#### Median pain intensity 30 min post-treatment

**Fig. 3:**
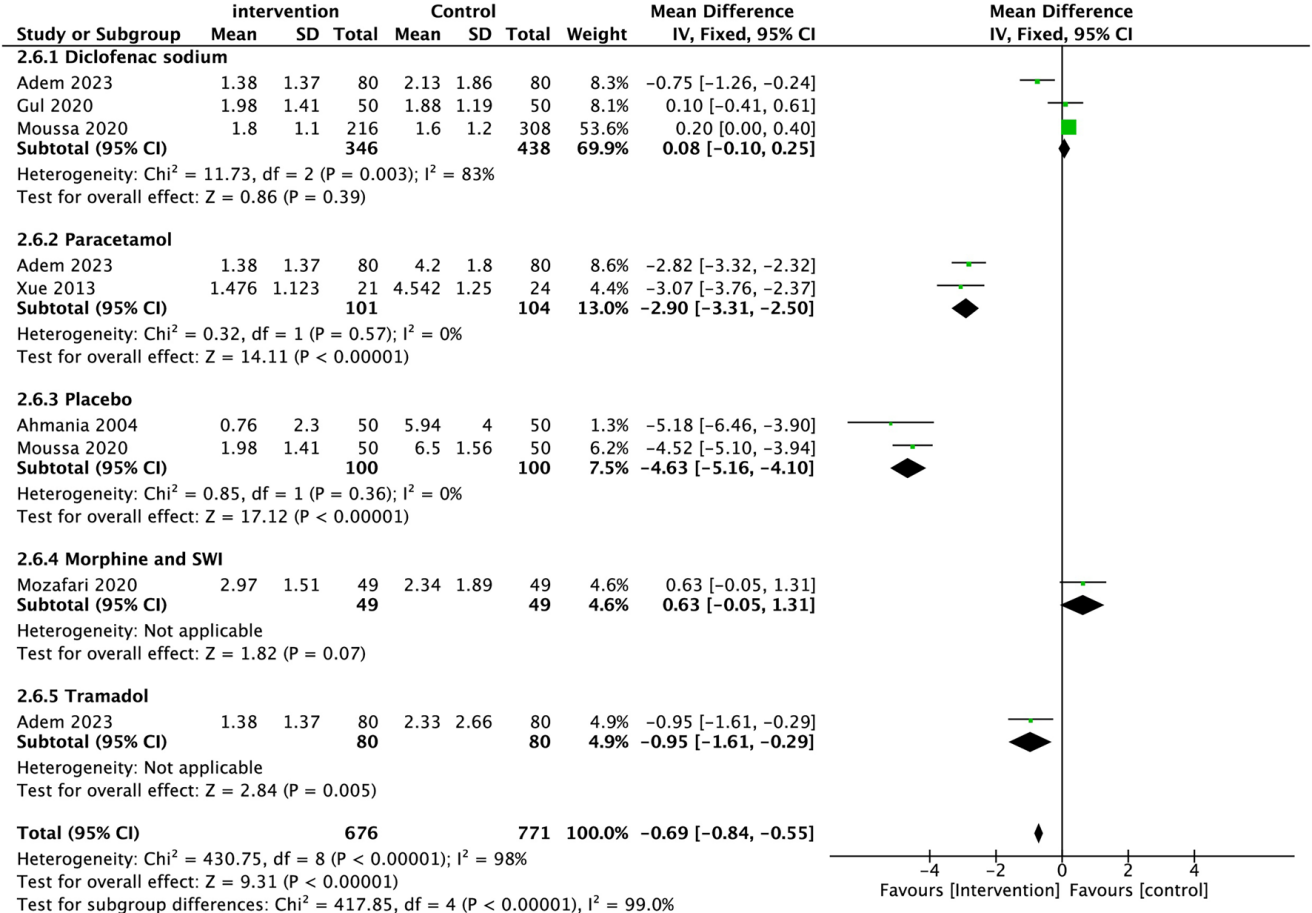
Forest plots of comparison of Sterile water injections against Diclofenac Sodium, Paracetamol, Placebo, Morphine with sterile water, Tramadol, Self-reported visual analogue scale (VAS) pain scores at 30 min after treatment. The solid squares denote the Mean difference (MDs). The horizontal lines represent the 95% confidence intervals (CIs), and the diamond denotes the pooled effect size

All six studies investigated the median pain intensity 30 min following intervention, with self-reported visual analogue scale (VAS) ranging from 0 to 10. Patients receiving SWI had the lowest pain score at this interval (1.74 ± 1.49), which was 9.1% lower than those receiving Diclofenac sodium (1.91 ± 1.53). Both Tramadol and Morphine groups exhibited an approximate 25.0% less analgesic effect than SWI, with a score of 2.33 ± 2.66 and 2.34 ± 1.89, respectively. Most notably, patients receiving paracetamol had a higher median pain score (4.27 ± 1.7) than those receiving SWI. Lastly, the group with the lowest analgesic effect at 30 min was the Sodium Chloride placebo group, reporting a 257.5% higher pain score than SWI (6.22 ± 3.03). Overall, the SWI groups demonstrated a lower median pain intensity 30 min post-treatment compared to control agents (MD = − 0.69, 95%CI: − 0.84, − 0.55, P < 0.001). Between-study heterogeneity was high (I^2^ = 98%, P < 0.001). (Fig. 3).

#### Sterile water injection compared to Diclofenac Sodium

Three studies compared SWI with diclofenac. In the Moussa et al. [21] trial, 50 patients received 75 mg of intramuscular Diclofenac sodium, and 50 received 0.5 mL of intracutaneous SWI at the most painful site. The pre-treatment and 30-min post-treatment median pain intensity expressed through a VAS of the two groups were similar. The SWI patients reported a reduction from 9.6 ± 0.61 to 1.98 ± 1.41, whilst the Diclofenac patients reported an improvement from 9.72 ± 0.64 to 1.88 ± 1.19. Four patients required rescue analgesia in the SWI group and seven in the Diclofenac group. These findings suggest that both SWI and Diclofenac sodium had similar effectiveness, with no significant difference in pain scores at 30 min (P = 0.702). Adverse events included nausea and vomiting in six patients receiving diclofenac and one patient receiving SWI; epigastric pain occurred in seven diclofenac cases, while injection site pain was reported in two diclofenac and one SWI patient. Adem et al. [20] compared two groups of 80 patients receiving the same drugs and doses as the above Moussa study. The VAS before injection was 8.66 ± 0.79 for the SWI group and 8.64 ± 0.70 for the Diclofenac group. At 30 min post-injection, the scores were 1.38 ± 1.37 after SWI and 2.13 ± 1.86 after Diclofenac (P = 0.016), with seven (8.8%) and nine (11.3%) patients requiring rescue analgesia, respectively. There were no adverse effects reported in the SWI group, but six (7.5%) in the Diclofenac group, which was significant (P = 0.013). Gul and Gul [22] used 2–3 mL of intracutaneous SWI and compared it against 75 mg of Diclofenac intramuscularly in groups of 216 and 308 renal colic patients, respectively. Whilst no explanation was provided for the significant difference in sample sizes, there was no significant difference in baseline characteristics between the two groups. The SWI group’s VAS before intervention was 6.4 ± 2.9, and the Diclofenac group was 6.6 ± 3.2. At 30 min after intervention, these decreased to 1.8 ± 11 and 1.6 ± 1.2, respectively. The difference between groups was not significant (P = 0.397). Three patients receiving SWI required rescue analgesia, and four received Diclofenac (P = 0.272).

#### Sterile water injection compared to Placebo

Two studies compared SWI to normal saline 0.9% placebo. Ahmadnia et al. [23] compared 50 patients receiving 0.5 mL of SWI and 50 patients receiving 0.5 mL of normal saline. The VAS score before injection was 9.86 ± 3 in the SWI group and 9.96 ± 19 in the placebo group, reducing to 0.79 ± 2.3 and 5.94 ± 4 respectively following administration (P < 0.001). Three patients in the SWI group required rescue analgesia versus 17 in the sodium chloride placebo group. Moussa et al. [21] also compared 0.5 cm^3^ of SWI to 0.5 cm^3^ of normal saline, with 50 patients in each group. The SWI group had a reduction in their pain score from 9.6 ± 0.61 to 1.98 ± 1.41 following injection, and the placebo group reduced from 9.20 ± 0.89 to 6.5 ± 1.56, with the significant values not reported in this case. The use of rescue analgesia was significantly lower in the SWI group, with four patients requiring it, compared to 47 in the saline placebo group. Patients in the placebo group had an 86.0% higher likelihood of requiring rescue analgesia within one hour (P < 0.001).

#### Sterile water injection compared to Morphine

A single study by Mozafari et al. [25] compared 0.5 mL of intradermal SWI to intravenous 0.1 mg/kg morphine combined with 0.5 mL of intradermal sterile water, administered over two minutes. Both groups contained 49 patients. Abstract data from this study were used, as the full-text article could not be accessed, nor could the authors be contacted. The VAS before treatment were 8.1 ± 1.26 for SWI and 9.46 ± 1.0 for the Morphine/SWI group; scores 30 min after treatment were 2.97 ± 1.51 and 2.34 ± 1.89 respectively (P = 0.035). No use of rescue analgesia was reported in either group, although itching and nausea were more commonly reported in the SWI group.

#### Sterile water injection compared to Paracetamol

Two studies compared SWI to paracetamol. Xue et al. [24] investigated the efficacy of SWI and paracetamol in pregnant women with renal colic using 0.5 mL of intracutaneous SWI (n = 21 patients) or 1 g of oral paracetamol (n = 24 patients). The pre-intervention VAS for the SWI group was 9 ± 1.17 whilst 8.54 ± 1.06 in the paracetamol group. Following treatment, these reduced to 1.47 ± 1.12 and 4.54 ± 1.25 respectively (P < 0.001). One woman in the SWI group required rescue analgesia, compared to 8 in the paracetamol group. Adem et al. [20] compared 0.5 mL of intracutaneous SWI to 1 g of intravenous paracetamol (both groups contained 80 patients). 30 min after administration, the VAS reduced from 8.66 ± 0.79 to 1.38 ± 1.37 in the SWI group, and from 8.68 ± 0.85 to 4.20 ± 1.8 in the paracetamol group (p < 0.000). The use of rescue analgesia was lower in the SWI group (7) than in the paracetamol group (15). No patients experienced adverse events in the SWI group, but two (2.5%) experienced adverse events in the paracetamol group (P = 0.129).

#### Sterile water injection compared to Tramadol

The Adem study [20] compared 0.5 mL of intracutaneous SWI to 100 mg of Intravenous Tramadol in groups of 80 patients. The VAS pain score before the intervention was 8.66 ± 0.79 for the SWI patients and 8.64 ± 0.72 for the Tramadol patients. These reduced to 1.38 ± 1.37 and 2.33 ± 2.66, respectively (P = 0.019). Rescue analgesia was used by seven patients receiving SWI and 17 of those receiving Tramadol. There was a significant difference in adverse events between the two groups; none experienced in the SWI group compared to 12 (15.0%) in the tramadol group (P = 0.003).

## Discussion

This systematic review and meta-analysis of randomised studies evaluating the use of sterile water injections for pain relief in acute renal colic has yielded potential clinically relevant findings for the management of this condition. Importantly, across the included studies, there were no significant differences in demographics or pain scores at presentation between the groups investigated. When comparing the analgesic effect of SWI to the other studied agents, there was a lower rate of rescue analgesia use and a lower self-reported pain score at 30 min post-treatment. Overall, heterogeneity varied considerably between outcomes, warranting caution in interpreting the findings. While the number of trials is low and the quality of evidence is limited, the available studies suggest that SWIs could play a role in the management of acute renal colic.

Several mechanisms have been proposed to explain the pain-relieving effects of SWIs. First, the Diffuse Noxious Inhibitory Control Theory describes that pain from distant body parts suppresses the pain in the spinal cord’s dorsal horn neurons, using pain to alleviate pain [26, 27]. Second, SWIs can cause skin irritation through osmotic changes caused by the water injection. This leads to the release of endogenous opioids, suppressing the perception of pain at peripheral nerve terminals [28]. Third, the Gate Control Theory, whereby first-order afferent nociceptors and low-threshold afferent mechanoreceptors converge in the same neurons in the substantia nigra. The pressure elicited by the SWI in the intradermal space activates mechanoreceptors, which further inhibit the nociceptive signals, opening a ‘gate’ to allow the transmission of non-nociceptive pain to the brain. By this mechanism, SWIs inhibit the transmission of painful stimuli to the brain and allow rapid pain control within minutes [29].

NSAIDs, paracetamol, and, to a lesser extent, opioids are the preferred analgesics in managing renal colic pain, according to the 2022 European Association of Urology guidelines [30]. While these agents provide varying degrees of pain relief, they may all cause adverse effects. For example, NSAIDs commonly cause GI side effects, nausea, vomiting, dizziness and impaired renal function, whilst opioids can cause itching, hallucinations, sedation, respiratory depression, dependence and hypotension [30]. A meta-analysis of the efficacy of SWIs, NSAIDs and opiates in renal colic concluded that all three had equivalent analgesic effects at 30 min after delivery [8]. However, adverse effects and the need for rescue analgesia were reportedly less in the NSAID group [8]. Our findings align with these conclusions. A larger number of adverse effects and a higher rate of rescue analgesia were reported in the paracetamol and Opioid groups compared to the NSAID group, as well as higher 30-min pain scores. Importantly, those receiving SWIs reported no adverse effects, except that of injection site pain, a lower rate of rescue analgesia, and lower pain scores at 30 min than those in the NSAID groups. These results suggest that SWI may be safer and more effective than currently widely used analgesic options in the treatment of renal colic, although the quality of the evidence is limited.

SWIs have been investigated in clinical settings where standard analgesia is contraindicated, such as during pregnancy [14, 31], where NSAIDs and opiate side effects include spontaneous abortion, congenital heart defects, neurological deficits, stillbirth and teratogenic risks [32]. Their potential has recently been recognised by the Nation Institute for Health and Care Excellence (NICE) for labour-related back pain [15]; however, this recommendation remains cautious due to inconsistent and low-quality evidence, concerns over unblinding and reports of significant injection discomfort. Additionally, patient-reported effectiveness was mixed, with some studies noting limited long-term pain relief and reluctance to undergo repeat injections [33]. In our review, SWI demonstrated superior analgesic effects compared to control agents, with greater patient-reported pain reduction and fewer patients requiring rescue analgesia, supporting their potential use. However, further high-quality research is needed to clarify their long-term efficacy and tolerability.

This study has clinical implications that warrant consideration. SWIs offer a low-cost, easily administered analgesic option with no risk of overdose and minimal side effects beyond injection site pain. Their safety profile makes them particularly useful for patients with contraindications to NSAIDs, such as those with renal impairment or during pregnancy. Given their broad availability, SWIs could be an accessible option in emergency settings. However, before clinical adoption, further research is required to standardise dosing, optimize administration techniques, and confirm their efficacy.

This systematic review has multiple strengths. Firstly, it adhered to PRISMA guidelines, ensuring a structured and transparent methodology. Secondly, to minimise bias, abstract and full-text screening, as well as data extraction, were independently conducted by two reviewers. Thirdly, the search strategy was comprehensive, incorporating all relevant synonyms and validated by experts in urology and systematic reviews. Lastly, major databases, including MEDLINE, EMBASE, and Web of Science, were searched to ensure all relevant trials were identified.

This study has several limitations. First, there was a low number of eligible RCTs and participants, which increases the risk of type 2 error. Second, there were concerns over bias in some studies, with many trials exhibiting a high or unclear bias in relation to the blinding of participants and clinicians. Third, inconsistencies in SWI administration protocols contributed to the overall heterogeneity. Finally, incomplete reporting of baseline characteristics and outcome measures may limit the confidence of our overall findings.

Our study has identified several areas for future research. Given the number of RCTs and the number of patients included, more RCTs could be conducted employing larger sample sizes to minimise the uncertainty in our findings. The trials could be more robust in design to minimise the risk of bias through the blinding process. Whilst all studies relied on radiological evidence for definitive confirmation of renal and ureteric stones, the modality varied between US and CT, and no standardised criteria for stone size were imposed. Future studies should establish clearer diagnostic criteria for renal colic. Lastly, while most studies used 0.5 mL intracutaneous SWI at the most painful point, this was not consistent across all studies. Future research should aim for a clear consensus on the optimal dose and injection site for SWIs.

## Conclusions

This systematic review and meta-analysis of randomised trials supports that SWIs can provide analgesia in renal colic, providing comparable patient-reported pain relief to Diclofenac and potentially superior pain relief compared to Morphine, but with a significantly lower side effect profile than both. While these findings are promising, the number and quality of eligible studies necessitate high-quality studies, including RCTs, before we definitively assert that SWIs should become part of standard clinical practice for analgesia in renal colic.

## Supplementary Information

Below is the link to the electronic supplementary material.

Supplementary file1 (DOCX 633 KB)

## Data Availability

No datasets were generated or analysed during the current study.

## References

[1] Holdgate A, Pollock T (2004) Systematic review of the relative efficacy of non-steroidal anti-inflammatory drugs and opioids in the treatment of acute renal colic. BMJ 328(7453):1401. 10.1136/bmj.38119.581991.55 15178585 10.1136/bmj.38119.581991.55 PMC421776

[2] Frassetto L, Kohlstadt I (2011) Treatment and prevention of kidney stones: an update. Am Fam Physician 84(11):1234–1242 22150656

[3] Scales CD, Smith AC, Hanley JM et al (2012) Prevalence of kidney stones in the United States. Eur Urol 62(1):160–165. 10.1016/j.eururo.2012.03.052 22498635 10.1016/j.eururo.2012.03.052 PMC3362665

[4] López M, Hoppe B (2010) History, epidemiology and regional diversities of urolithiasis. Pediatr Nephrol 25(1):49–59. 10.1007/s00467-008-0960-5 21476230 10.1007/s00467-008-0960-5 PMC2778769

[5] Monga M, Roudakova K (2014) The evolving epidemiology of stone disease. Indian J Urol 30(1):44. 10.4103/0970-1591.124206 24497682 10.4103/0970-1591.124206 PMC3897053

[6] Aune D, Mahamat-Saleh Y, Norat T et al (2018) Body fatness, diabetes, physical activity and risk of kidney stones: a systematic review and meta-analysis of cohort studies. Eur J Epidemiol 33(11):1033–1047. 10.1007/s10654-018-0426-4 30066054 10.1007/s10654-018-0426-4 PMC6208979

[7] Wijarnpreecha K, Lou S, Panjawatanan P et al (2018) Nonalcoholic fatty liver disease and urolithiasis: a systematic review and meta-analysis. J Gastrointest Liver Diseases. 10.15403/jgld.2014.1121.274.nac 10.15403/jgld.2014.1121.274.nac 30574625

[8] Pathan SA, Mitra B, Cameron PA (2018) A systematic review and meta-analysis comparing the efficacy of nonsteroidal anti-inflammatory drugs, opioids, and paracetamol in the treatment of acute renal colic. Eur Urol 73(4):583–595. 10.1016/j.eururo.2017.11.001 29174580 10.1016/j.eururo.2017.11.001

[9] Tsiotras A, Smith RD, Pearce I et al (2017) British association of Urological Surgeons standards for management of acute ureteric colic. J Clin Urol 11(1):58–61. 10.1177/2051415817740492

[10] Bhala N, Emberson J, Merhi A et al (2013) Vascular and upper gastrointestinal effects of non-steroidal anti-inflammatory drugs: meta-analyses of individual participant data from randomised trials. Lancet 382(9894):769–779. 10.1016/s0140-6736(13)60900-9 23726390 10.1016/S0140-6736(13)60900-9 PMC3778977

[11] Welk BK, Teichman JM (2007) Pharmacological management of renal colic in the older patient. Drugs Aging 24(11):891–900. 10.2165/00002512-200724110-00002 17953457 10.2165/00002512-200724110-00002

[12] Alelign T, Petros B (2018) Kidney stone disease: an update on current concepts. Adv Urol 2018:1–12. 10.1155/2018/3068365 10.1155/2018/3068365 PMC5817324 29515627

[13] di Maio G (1949) Interception of renal colic by intradermal injection of twice-distilled water at the pain points. Minerva Med 40(43 Pt 2):265–268 (**PMID:15391576**) 15391576

[14] Lee N, Gao Y, Collins SL et al (2020) Caesarean delivery rates and analgesia effectiveness following injections of sterile water for back pain in labour: a multicentre, randomised placebo-controlled trial. EClinicalMedicine 25:100447. 10.1016/j.eclinm.2020.100447 32954233 10.1016/j.eclinm.2020.100447 PMC7486301

[15] Evidence reviews for sterile water injections: Intrapartum care: Evidence review C. PubMed. London: National Institute for Health and Care Excellence (NICE); 2023 (NICE Guideline, No. 235). Available from: https://www.ncbi.nlm.nih.gov/books/NBK596273/ [Last accessed: March 10, 2025] 37856631

[16] Page MJ, McKenzie JE, Bossuyt PM et al (2021) The PRISMA 2020 statement: an updated guideline for reporting systematic reviews. British Med J. 10.1136/bmj.n71 10.1136/bmj.n71 PMC8005924 33782057

[17] Sterne JAC, Savovic J, Page MJ et al (2019) RoB 2: a revised tool for assessing risk of bias in randomised trials. BMJ 366(1):l4898. 10.1136/bmj.l4898 31462531 10.1136/bmj.l4898

[18] Cumpston M, Li T, Pagge MJ et al (2021) Updated guidance for trusted systematic reviews: a new edition of the Cochrane Handbook for systematic reviews of Interventions. Cochrane Database Syst Rev 10(4):565 10.1002/14651858.ED000142 PMC10284251 31643080

[19] Hozo SP, Djulbegovic B, Hozo I (2005) Estimating the mean and variance from the median, range, and the size of a sample. BMC Med Res Methodol 5(1):13. 10.1186/1471-2288-5-13 15840177 10.1186/1471-2288-5-13 PMC1097734

[20] Az A, Sogut O, Akdemir T et al (2024) Intradermal sterile water injection: safe and effective alternative for relief of acute renal colic in the emergency department. J Emerg Med 66(2):83–90. 10.1016/j.jemermed.2023.10.014 38267297 10.1016/j.jemermed.2023.10.014

[21] Moussa M, Papatsoris AG, Chakra MA (2021) Intradermal sterile water injection versus diclofenac sodium in acute renal colic pain: a randomized controlled trial. Am J Emerg Med 44:395–400. 10.1016/j.ajem.2020.04.079 32444296 10.1016/j.ajem.2020.04.079

[22] Gul A, Gul M (2019) Intracutaneous sterile water injection for pain relief during extracorporeal shock wave lithotripsy: comparison with diclofenac sodium. Urolithiasis 48(2):103–108. 10.1007/s00240-019-01147-9 31278470 10.1007/s00240-019-01147-9

[23] Ahmadnia H, Rostami Y (2004) Treatment of renal colic using intracutaneous injection of sterile water. Urol J 1(3):200–203 (**PMID:17914689**) 17914689

[24] Xue P, Tu C, Wang K et al (2013) Intracutaneous sterile water injection versus oral paracetamol for renal colic during pregnancy: a randomized controlled trial. Int Urol Nephrol 45(2):321–325. 10.1007/s11255-013-0405-3 23443875 10.1007/s11255-013-0405-3

[25] Mozafari J, Verki MM, Tirandaz F et al (2020) Comparing intradermal sterile water with intravenous morphine in reducing pain in patients with renal colic: a double-blind randomized clinical trial. Rev Recent Clin Trials 15(1):76–82. 10.2174/1574887114666191118153600 31738150 10.2174/1574887114666191118153600

[26] Le Bars D, Dickenson AH, Besson JM (1979) Diffuse noxious inhibitory controls (DNIC). I. Effects on dorsal horn convergent neurones in the rat. Pain 6(3):283–304. 10.1016/0304-3959(79)90049-6 460935 10.1016/0304-3959(79)90049-6

[27] Morgan MM, Whitney PK (1996) Behavioral analysis of diffuse noxious inhibitory controls (DNIC): antinociception and escape reactions. Pain 66(2):307–312. 10.1016/0304-3959(96)03061-8 8880854 10.1016/0304-3959(96)03061-8

[28] Derry S, Straube S, Moore RA et al (2012) Intracutaneous or subcutaneous sterile water injection compared with blinded controls for pain management in labour. Cochrane Database Syst Rev 1:CD009107. 10.1002/14651858.CD009107.pub2 22258999 10.1002/14651858.CD009107.pub2 PMC11663508

[29] Ropero Peláez FJ, Taniguchi S (2015) The gate theory of pain revisited: modeling different pain conditions with a parsimonious neurocomputational model. Neural Plast 1:1–14. 10.1155/2016/4131395 10.1155/2016/4131395 PMC4814802 27088014

[30] Skolarikos A, Geraghty R, Somani B et al (2025) European association of urology guidelines on the diagnosis and treatment of urolithiasis. Eur Urol 88(1):64–75. 10.1016/j.eururo.2025.03.011 40268592 10.1016/j.eururo.2025.03.011

[31] Fogarty V (2008) Intradermal sterile water injections for the relief of low back pain in labour—a systematic review of the literature. Women Birth 21(4):157–163. 10.1016/j.wombi.2008.08.003 18926789 10.1016/j.wombi.2008.08.003

[32] Li DK, Liu L, Odouli R (2003) Exposure to non-steroidal anti-inflammatory drugs during pregnancy and risk of miscarriage: population based cohort study. BMJ 327(7411):368. 10.1136/bmj.327.7411.368 12919986 10.1136/bmj.327.7411.368 PMC175811

[33] Muraca GM, Kramer JLK, Butwick AJ (2024) Sterile water injections for back pain in labour. BMJ 385:q1187. 10.1136/bmj.q118 38830694 10.1136/bmj.q1187

