# Supplementary material for: Sterile water injections for analgesia in renal colic: a meta-analysis of level 1 evidence
Source: World J Urol. 2025 Sep 16;43(1):557. doi: 10.1007/s00345-025-05920-x (PMC12441071; doi:10.1007/s00345-025-05920-x)

**Supplementary material**

**Title Page**

Title: Sterile water injections for analgesia in renal colic: a meta-analysis of Level 1 evidence

Journal name: World Journal of Urology

Authors: Ioannis Perros^1#^, Balamrit Singh Sokhal*^1,2#^, Christopher Swift^3^, Mark Kitchen^1,2^, Christian Mallen^1^, Bhaskar Somani^4^

1.    School of Medicine, Keele University, Keele, UK

2. Department of Urology, University Hospital of North Midlands, Stoke-On-Trent, UK

3. Wirral University Hospital, Liverpool, UK

4. Department of Urology, University Hospital Southampton, Southampton, UK

#Joint first authors

Contact emails and ORCIDs:

Ioannis Perros: [ioannisperros@outlook.com](mailto:ioannisperros@outlook.com) ; ORCID: 0000-0001-7529-5497

Balamrit Singh Sokhal: [balamritsokhal@gmail.com](mailto:balamritsokhal@gmail.com); ORCID: 0000-0002-2315-0744

Christopher Swift: [christopher.swift1@nhs.net](mailto:christopher.swift1@nhs.net); ORCID: 0009-0006-8762-874X

Mark Kitchen, [m.o.kitchen@keele.ac.uk](mailto:m.o.kitchen@keele.ac.uk); ORCID: N/A

Christian Mallen: [c.d.mallen@keele.ac.uk](mailto:c.d.mallen@keele.ac.uk); ORCID: 0000-0002-2677-1028

Bhaskar Somani: [bhaskarsomani@yahoo.com](mailto:bhaskarsomani@yahoo.com);  ORCID: 0000-0002-6248-6478

**Supplementary Table S1.** Example search strategy

| **#** | **Search*** | **Results**  **18/10/23** |
| --- | --- | --- |
| 1 | “Renal Colic”.sh. | 691 |
| 2 | “Urolithiasis+”.sh. | 3,119 |
| 3 | ((renal* or kidney* or urethra* or urinary) adj2 (colic* or stone* or calculi*)).ti or ((renal* or kidney* or urethra* or urinary) adj2 (colic* or stone* or calculi*)).ab. | 30,560 |
| 4 | (urolithiasis or nephrolithiasis or urinary lithiasis or ureterolithiasis).ti or (urolithiasis or nephrolithiasis or urinary lithiasis or ureterolithiasis).ab. | 15,378 |
| 5 | ((flank or loin) adj1 pain).ti. or ((flank or loin) adj1 pain).ab. | 4,825 |
| 6 | 1 or 2 or 3 or 4 or 5 | 44,813 |
| 7 | ((water or sterile or saline or chloride) adj5 (inject* or subcut* or intradermal or intracut* or intramusc*)).ti. or ((water or sterile or saline or chloride) adj5 (inject* or subcut* or intradermal or intracut* or intramusc*)).ab. | 40,319 |
| 8 | Randomized controlled trial +sh. | 600,990 |
| 9 | Random* control* trial*.ti. or Random* control* trial*.ab. | 266,145 |
| 10 | 8 or 9 | 771,092 |
| 11 | 6 and 7 and 10 | 20 |
| 12 | (Animals+ not Humans).sh. | 5,127,352 |
| 13 | (((“Renal Colic” or “Urolithiasis” or (((renal* or kidney* or ureter* or urethra* or urinary) adj2 (colic* or stone* or calcul*)) or ((renal* or kidney* or ureter* or urethra* or urinary) adj2 (colic* or stone* or calculi*))) or (urolithiasis or nephrolithiasis or urinary lithiasis or ureterolithiasis or (urolithiasis or nephrolithiasis or urinary lithiasis or ureterolithiasis)) or (((flank or loin) adj1 pain) or ((flank or loin) adj1 pain))) and (((water or sterile or saline or chloride) adj5 (inject* or subcut* or intradermal or intracut* or intramusc*)) or ((water or sterile or saline or chloride) adj5 (inject* or subcut* or intradermal or intracut* or intramusc*))) and (Randomized controlled trial+ or (Random* control* trial* or Random* control* trial*))) not (Animals+ not Humans)).af. | 20 |

^*This search strategy was adapted for PubMed.^

**Supplementary Table S2**. Sterile water injection protocol

| **Study** | **Sterile water injection** | | | |
| --- | --- | --- | --- | --- |
|  | **Route of administration** | **Volume per injection** | **Number of injections** | **Site Selection** |
| **Adem et al.** | Intradermal | 0.5mL | 4 | Most painful point in the flank |
| **Mousa et al.** | Intracutaneous | 0.5cm^3^ | NP | Most painful point in the flank |
| **Gul et al.** | Intracutaneous | 2-3mL | 1 | Most painful point in the flank |
| **Ahmadnia et al.** | Intracutaneous | 0.5mL | 1 | Most painful point in the flank |
| **Mozafari et al.** | Intradermal | 0.5mL | 1 | Most painful point in the flank or the centre of the most painful area |
| **Xue et al.** | Intradermal | 0.5mL | 1 | Most painful point in the flank or the centre of the most painful area |

**^Abbreviations: NP, not provided^**

**Supplementary Fig S1.** Risk of bias summary and graph showing authors’ judgments about each risk of bias


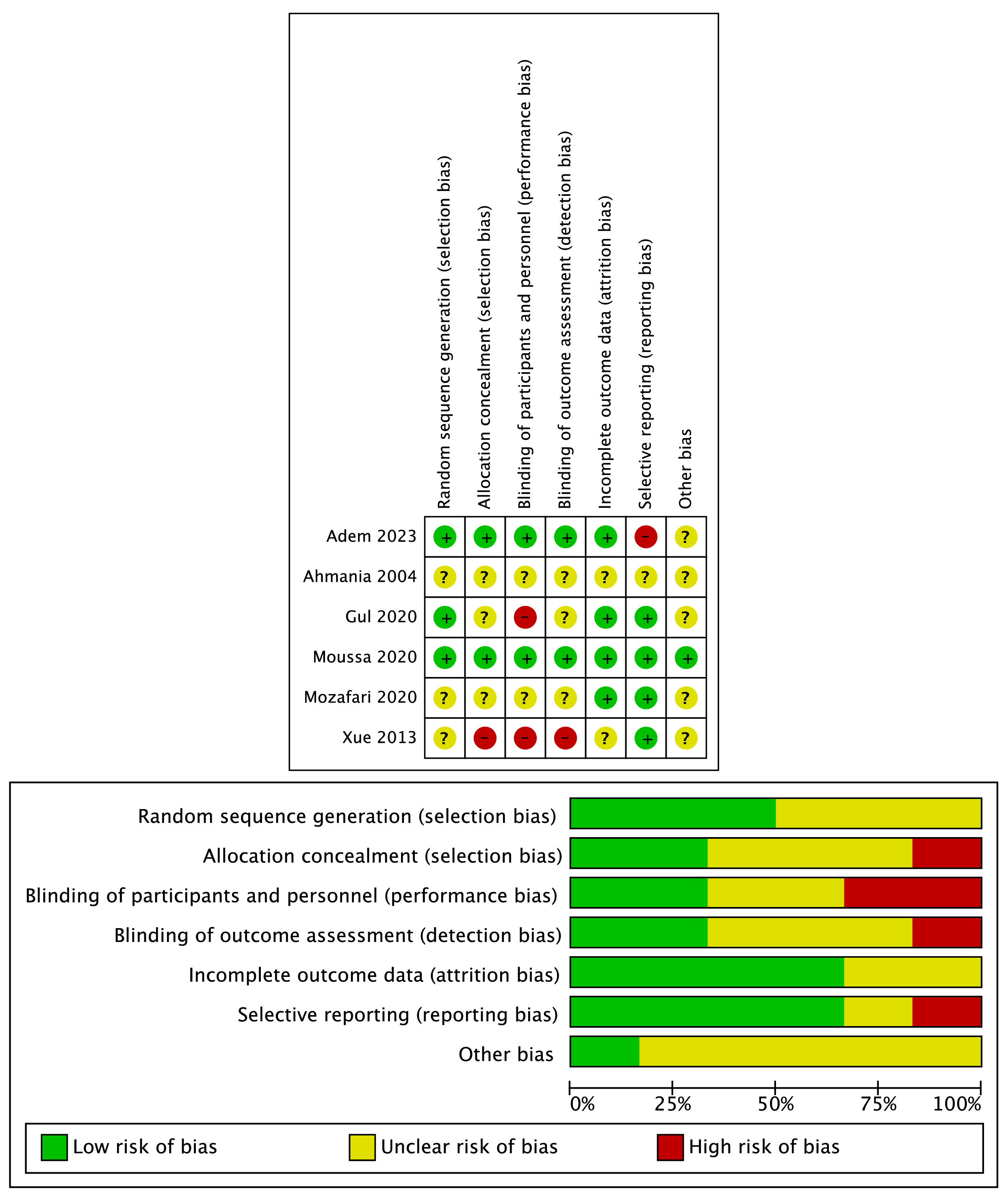


**Visual abstract**


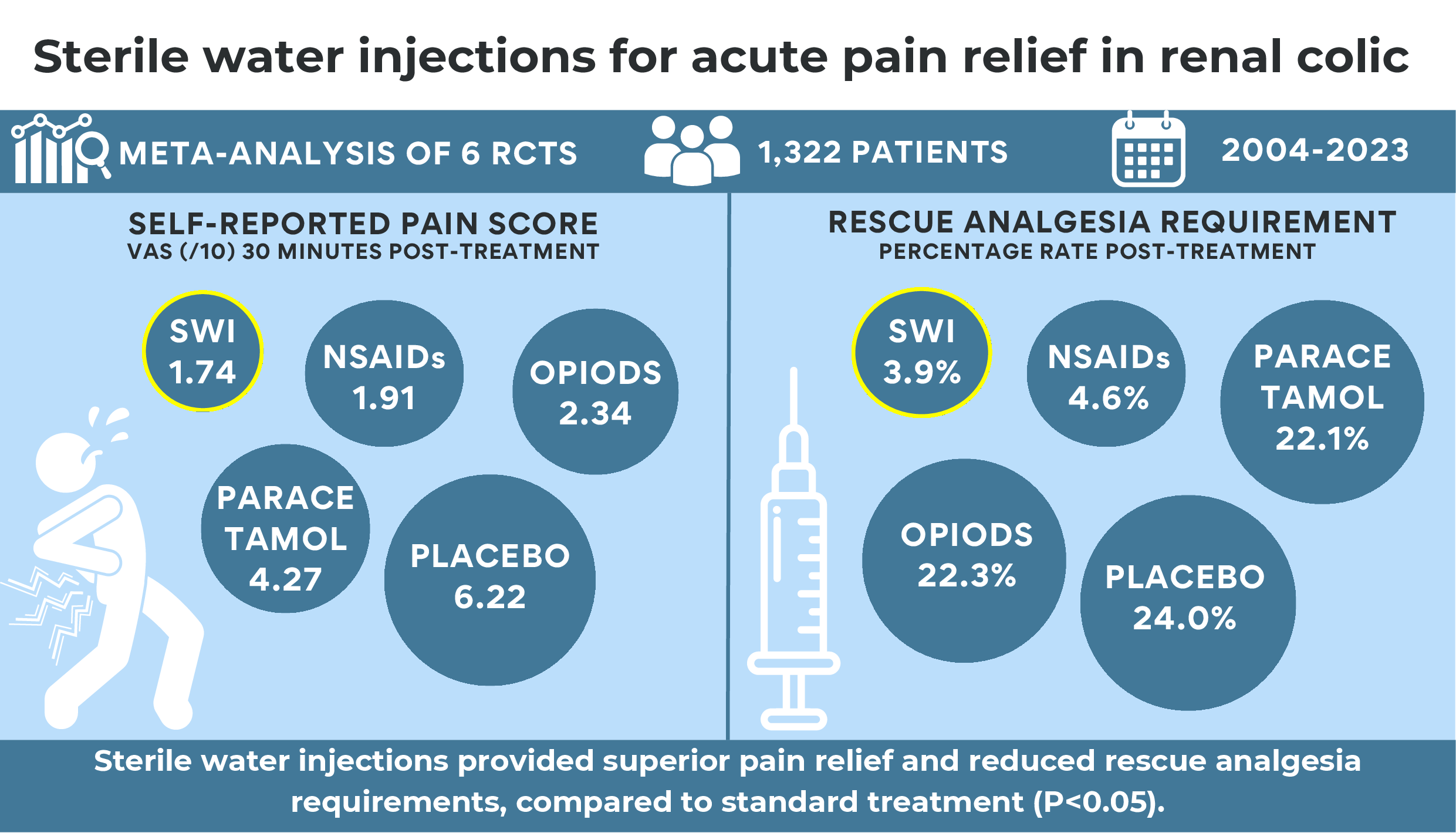

Supplement: Supplementary file 1 — Supplementary file1 (DOCX 633 KB) [file 345_2025_5920_MOESM1_ESM.docx]
